# Variants in Candidate Genes for Phenotype Heterogeneity in Patients with the 22q11.2 Deletion Syndrome

**DOI:** 10.1155/2024/5549592

**Published:** 2024-03-30

**Authors:** Natalia Nunes, Beatriz Carvalho Nunes, Malú Zamariolli, Diogo Cordeiro de Queiroz Soares, Leonardo Caires dos Santos, Anelisa Gollo Dantas, Vera Ayres Meloni, Sintia Iole Belangero, Vera Lúcia Gil-Da-Silva-Lopes, Chong Ae Kim, Maria Isabel Melaragno

**Affiliations:** ^1^Genetics Division, Department of Morphology and Genetics, Universidade Federal de São Paulo, São Paulo, Brazil; ^2^Genetics Unit, Instituto da Criança, Universidade de São Paulo, São Paulo, Brazil; ^3^Department of Translational Medicine, School of Medical Sciences, University of Campinas, Campinas, São Paulo, Brazil

## Abstract

22q11.2 deletion syndrome (22q11.2DS) is a microdeletion syndrome with a broad and heterogeneous phenotype, even though most of the deletions present similar sizes, involving ∼3 Mb of DNA. In a relatively large population of a Brazilian 22q11.2DS cohort (60 patients), we investigated genetic variants that could act as genetic modifiers and contribute to the phenotypic heterogeneity, using a targeted NGS (Next Generation Sequencing) with a specific Ion AmpliSeq panel to sequence nine candidate genes (*CRKL*, *MAPK1*, *HIRA*, *TANGO2*, *PI4KA*, *HDAC1*, *ZDHHC8*, *ZFPM2*, and *JAM3*), mapped in and outside the 22q11.2 hemizygous deleted region. *In silico* prediction was performed, and the whole-genome sequencing annotation analysis package (WGSA) was used to predict the possible pathogenic effect of single nucleotide variants (SNVs). For the *in silico* prediction of the indels, we used the genomic variants filtered by a deep learning model in NGS (GARFIELD-NGS). We identified six variants, 4 SNVs and 2 indels, in *MAPK1*, *JAM3*, and *ZFPM2* genes with possibly synergistic deleterious effects in the context of the 22q11.2 deletion. Our results provide the opportunity for the discovery of the co-occurrence of genetic variants with 22q11.2 deletions, which may influence the patients´ phenotype.

## 1. Introduction

The 22q11.2 deletion syndrome (22q11.2DS) results from the loss of chromosome 22 DNA segments and is the most frequent microdeletion syndrome. The presence of blocks of repetitive and similar DNA (low copy repeats, LCRs) in the 22q11.2 region predisposes to nonallelic homologous recombination, resulting in greater instability in this genomic region [1]. Most of these deletions (∼90%) comprise 3 Mb between the LCR22A and LCR22D, involving around 60 genes [1, 2]. The manifestation of the clinical signs of 22q11.2DS is broad and may affect different organs and systems, ranging from mild to severe. Even though most patients have similar-sized deletions, it can result in variable phenotypes, even in cases of inherited deletions or monozygotic twins [3–5]. 22q11.2DS phenotypes include congenital cardiac malformations, velopharyngeal dysfunction, metabolic and immunological disorders, and behavioral and cognitive difficulties, with an increased incidence of depressive anxiety, attention disorders, and schizophrenia [1, 4, 6, 7]. It has already been shown that genes outside the deleted region might be associated with psychiatric, cardiac, and immunophenotypes in the 22q11.2DS [8, 9]. One of the hypotheses lies in the fact that allelic variation, such as SNVs and indels, within the nondeleted 22q11.2 allele or in other genes mapped outside the 22q11.2 region, could influence the phenotype outcome of the 22q11.2DS [10]. Driven by this hypothesis, the whole-genome sequencing of patients with 22q11.2DS gave indications that the *HADC1* (Histidine Decarboxylase) and *ZFPM2* (Zinc Finger Protein, FOG Family Member 2) genes may act as genetic modifiers associated with cardiac defects [11–13]. Changes in the immune system, such as inflammation and T-cell-mediated immune response, were shown to be associated with schizophrenia [14–16]. Garber et al. [17] saw that a subpopulation of T-cells, the Th17 cells, influenced the development and/or regulation of psychotic symptoms in 22q11.2DS. A combined dysfunction of the relationship between the *MAPK1* (Mitogen-Activated Protein Kinase 1) and *CRKL* (Like Proto-Oncogene, Adaptor Protein) genes also appears to be related to the syndrome's phenotypic variability. In particular, *CRKL* appears to be involved in the occurrence of cardiac abnormalities, mostly tetralogy of Fallot [6, 9, 18, 19]. Still, the genetic analysis of 22q11DS remains highly elusive and is complicated by the complex regulatory circuits of early embryonic formation as well as by phenotypic heterogeneity [20, 21]. The advances in NGS (Next Generation Sequencing) have significantly increased the possibilities of genetic analysis in general, improving the chance of detecting gene variants in a substantial proportion of patients.

Studies in the literature show that some genes in hemizygosity must contribute to the phenotype of patients. Hestand et al. [22] performed candidate gene sequencing in the 22q11.2 region in 127 patients. They suggested that nonsynonymous variants found in several genes associated with the syndrome's phenotypes, including *PI4KA* (Phosphatidylinositol 4-Kinase Alpha), could result in partially functional proteins [23–25]. In addition, the *HIRA* gene (Histone Chaperone Complex) was indicated to be necessary for efficient suppression of viral infection, being involved in the chromatinization of viral DNA, and participating in intrinsic antiviral immunity [26].

Targeted NGS with gene panels offers a unique opportunity to sequence multiple genes at a lower cost and with less effort and thus is an efficient tool for mutation screening in the clinical diagnostic setting [27, 28]. This approach was used by Heike et al. [29] to sequence the TBX1 region in patients with 22q11.DS to identify genetic variants in *TBX1* that could influence the phenotype, and by Pulignani et al. [30] to sequence the *ZFPM2* gene in patients with nonsyndromic congenital heart defects. It is believed that the investigation of mechanisms influencing the 22q11.2DS phenotypic heterogeneity can help to understand the developmental pathways of the clinical traits involved and shed light on the management challenges of these patients [1, 31, 32]. Nevertheless, to date, no 22q11.2DS study based on targeted NGS was carried out in the Brazilian population, which is essential since the Brazilian population is mixed, whereby requires care to estimate the allelic frequencies of polymorphisms in a representative way and is a challenge considering only international databases, given that Latino populations are underrepresented. This is the first study that aimed to perform a targeted NGS of a specific gene panel in a Brazilian population of 22q11.2DS.

## 2. Methods

### 2.1. Sample

The sample of the present study consists of 60 patients with ∼3 Mb deletions in 22q11.2 (average age of 19 ± 3 years in the first clinical evaluation), recruited at the Medical Genetic Center of the Universidade Federal de São Paulo (UNIFESP), at the Child Institute of the Faculty of Medicine of the Universidade de São Paulo (FMUSP), and the University Hospital of the Universidade Estadual de Campinas (UNICAMP), all in Brazil. Parental analysis of the 22q11.2 deletion was performed for at least one parent for 27 patients. Among the sample of patients, cases with and without cardiac and immunological or psychiatric alterations were evaluated. The statistical power of the sample size was calculated using the tool G Power (Universität Düsseldorf, Germany), which resulted in a statistical power of 81% with an *α* of 0.02 and an OR >20.

### 2.2. Custom 22q11.2 Gene Panel for the Risk of Cardiac or Immune-Psychiatric Phenotypes

The choice of genes for the panel was performed based on the gene expression microarray data from a previous study carried out by our research group [8], additionally to the gathering information using the following online tools: PubMed [33], to consult the literature for articles and scientific reviews; GeneCards [34], to general aggregate data on the function and pathways in which the gene is involved; UCSC Genome Browser [35], to check the genomic coordinates, size, and number of exons and introns of the gene; and OMIM (Online Mendelian Inheritance in Man) [36], to find out if the gene has ever been linked to comorbidity. The following genes were selected for the study: *CRKL*, *MAPK1*, *HIRA*, *TANGO2*, *PI4KA*, *HDAC1*, *ZDHHC8*, *ZFPM2*, and *JAM3* (based on Table 1).

### 2.3. Targeted NGS

The gene panel was designed with the online tool Ion AmpliSeq Designer [37] to capture coding regions, splicing sites, and immediately adjacent intron sequences. The sequencing of selected genes was performed on the equipment Ion Torrent (Thermo Fisher). The construction of libraries was performed by the Ion AmpliSeq Library Kit 2.0–96 and quantified by the Ion Library Equalizer Kit (Thermo Fisher). The template was subjected to clonal amplification in micelles using the Hi-Q Ion OT Kit (Thermo Fisher) in Ion OneTouch 2 (Thermo Fisher). The template enrichment was performed on the Ion OneTouch ES (Thermo Fisher) and applied to the Ion 316 Chip (Thermo Fisher). The tools Ion Torrent Suite and Ion Reporter (Thermo Fisher) were used for the initial data analysis. The Ion Reporter and Torrent Suite software were initially used for sequence alignment, coverage number, and determination of samples´ genotypes.

### 2.4. Analysis of Variants and Filtering

The annotation and filtering of variants were performed from the VCF file (Variant Call Format) generated by the Ion Reporter tool (Thermo Fisher) in the UNIX environment. Variants with low sequencing coverage (<30x), according to [38], were excluded. Variants with minor allele frequency (MAF, Minor Allele Frequency) < 5% were selected from the gnomAD database [39] and the Brazilian reference cohort database with 609 human genome samples ABraOM (Brazilian Online Archive of Mutations) [40]. To assess the veracity of the identified indel, the GENOMIC VARIANTS FILTERING BY DEEP LEARNING MODELS IN NGS (GARFIELD-NGS) [41] was used, which rely on machine learning models to distinguish true positive from false positive indels call. To study the possible effect of the variants, *in silico* prediction was performed using the WGSA (Whole Genome Sequencing Annotation) analysis package according to Liu et al. [42]. The package has an annotation pipeline for human genome sequencing studies, aggregating databases, and prediction tools of various types; for the prediction of pathogenicity of variants, the databases and scores that were considered are shown in Supplementary Figure 1 (Supplementary Figure 1).

### 2.5. SNP Burden

The SNP-set (sequence) Kernel Association Test (SKAT) [43], based on Fisher's method, was used to examine whether all sequenced variants together contribute to the risk of cardiac or immunopsychiatric phenotypes.

### 2.6. Variants Validation

Sanger sequencing was performed to validate variants with pathogenic potential obtained from *in silico* analyses. The primers were designed for amplification reactions in the Primer3 program [44, 45], and the quality was verified by the OligoAnalyzer ™ Tool [46]. Amplification was performed using the PCR Master kit (Promega). The generated amplification products were purified with the QIAquick PCR Purification kit (Qiagen) and submitted to the sequencing reaction using the commercial kit BigDye version 3.1 (Applied Biosystems) and the Genetic Analyzer 3130xl equipment (Applied Biosystems).

## 3. Results

### 3.1. Parental Analysis

For 16 patients, parental analysis was performed for both parents; for 10 patients, it was performed only for the mother; and for one patient, it was performed only for the father. Among the patients for whom only the mother sample was available, three mothers presented the typical 3 Mb deletion in the 22q11.2 region. Two of them were part of the 60 patients included in this study. No other parent had deletions in the 22q11.2 region.

### 3.2. Phenotyping and SNV Burden

A total of 60 individuals with the canonical ∼3 Mb deletion between LCR22A and LDR22D were assessed for traditional 22q11.2 phenotypes. Our population of patients with 22q11.2DS exhibited cardiac and immunopsychiatric phenotypes consistent with previously published literature [1], being 32 (52%) patients with cardiac malformations and 28 (46%) patients with immunopsychiatric alterations.

Among these, 19 (31%) patients had both presented cardiac malformations together with immunopsychiatric alterations. The burden analysis (SKAT) did not show any association with the absolute number of variants sequenced in the genes and the phenotype groups assessed (cardiac malformations and immunopsychiatric alterations were compared with patients without cardiac alterations and without immunopsychiatric alterations, respectively) by Fisher's exact test (*p* < 0.05).

### 3.3. Variant's Prediction

**Figure 1:**
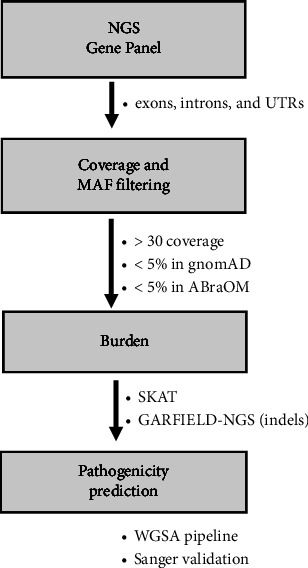
Data analysis pipeline.

A total of 2,923 variants were identified in the nine genes sequenced in 60 patients, with an average of 40 variants per patient, without the application of filters (Figure 1, Supplementary Table S1). All target genes had sequencing coverage above 85% of its extension (Supplementary Table S2). After the pipeline for filtering the variants with potential effects, four single nucleotide variants and two indel variants remained. Regarding SNVs, four variants were interpreted with a possible effect on the phenotype (Table 2): rs897688340, rs13058, rs41282607, mapped in *MAPK1*, and rs7936421, mapped in *JAM3* (Table 2). The coordinate identified is also a transcription factor binding site and a target for miRNA (MAPK1 miR-14303p). Two SNVs were predicted to be an eQTL and a transcription binding site. The variant rs897688340 is mapped at the UTR3 region of the *MAPK1* gene. It was predicted as potentially deleterious by CADD and FATHMM-XL, and it was also predicted to be likely to affect binding by RegulomeDB. The other two variants in this same gene (rs13058 and rs41282607) are registered at dbSNP by rs897688340 and rs41282607, respectively, and both are predicted as deleterious by the FATHMM-XL and CADD tools. Finally, the variant rs7936421, also associated in a genome-wide association study for cardiac valves, was the SNV in the *JAM3* gene, also located at an eQTL region, and predicted as deleterious by the CADD and FATHMM-XL tools. We also identified two indel variants that were highly predicted as pathogenic in all prediction tools consulted (Table 3). One of them, registered in the dbSNP with rs199956937 and mapped at the *ZFPM2* gene, was predicted to be at the target region of four miRNA (miR-130-3p; miR-17-5p; miR-143-5p; miR-340-5p). The other indel, identified in the *JAM3* gene, was registered in the dbSNP with rs3216140 and was predicted to be in the binding site of the transcription factor EZH2.

## 4. Discussion

In this study, we identified six genomic variants with possible effects on the phenotype of 22q11.2DS by a targeted NGS approach followed by appropriate filtering strategies. These variants will be discussed according to their prediction groups and the phenotypes of the patients in which they were identified (Table 2): [1] Expression Quantitative Trait Locus (eQTL), and Transcription Factor (TF) targets; [2] Hits on genomic association studies in a related phenotype (Table 2).

### 4.1. Expression Quantitative Trait Locus (eQTL) and Transcription Factor (TF) Targets

The analysis of the identified SNVs as potential eQTLs provided relevant results for three SNVs, whose genotypes could affect the expression of their related genes. The variants rs13058 and rs41282607 in *MAPK1* were found to be differentially associated in different tissues, including arteries, stomach, and thyroid, suggesting that these variants are likely to affect gene functions that are important for the body as a whole, which makes sense once they were identified in patients with cardiac malformations and immune-psychiatric alteration, respectively [47, 48]. We identified indels in the *ZFPM2* and *JAM3* genes that may be possibly pathogenic; they could be playing a role as a phenotype modifier in the patients. *ZFPM2* variants were found in three patients with cardiac malformations and had previously been associated with tetralogy of Fallot, a conotruncal heart defect commonly observed in patients with 22q11.2DS [12, 49]. The ZFPM2 protein acts as a cofactor for dosage-sensitive GATA transcription factors during embryonic heart development in mouse models, and it is speculated that variants in this gene could lead to conotruncal heart defects [12, 50]. Regarding the *JAM3*, this gene is one of the candidate genes of the cardiac phenotype in patients with 11q25 haploinsufficiency which is an analogous syndrome to the 22q11.2DS [51, 52]. The indel variant in *JAM3* was identified in the binding site of transcription factor EZH2 which controls the methylation of H3K27 histone and can also act through methylation of nonhistone proteins, being a potential mechanism for EZH2-mediated gene activation, the perturbation of this pathway can lead to cardiac defects and was already established as a driven mechanism in some types of cancer [53–55].

### 4.2. Hits on Genomic Association Studies in a Related Phenotype

The SNV in the *JAM3* gene (rs7936421), mapped in chromosome 11, found in 16 patients with diverse phenotypes, and even with a higher frequency in the population, is of interest. Since this same SNV has already been associated in an association study for the cardiac phenotype in the GRASP database, this database includes variants that have been significantly associated in genome-wide association studies [56]. It is known that SNVs in eQTL can influence mRNA expression levels [57]. That is the case of the rs793642, which was predicted to be at an eQTL region in the artery aorta and muscle-skeletal tissues according to the GTEX database. Most importantly, we identified the same variants in more than one patient, suggesting that the co-occurrence of two or more rare variants may have an additive or synergistic deleterious effect with the deletion in 22q11.2. Accordingly, we can speculate that each variant alone could be tolerated. Still, when combined with another genetic event, as with the deletion, it would lead to the difference of penetrance of some of the syndrome phenotypes [28, 58]. Hestand et al. [22] studied 127 individuals with the 22q11.2DS using next-generation sequencing to sequence the genes in the 22q11.2 region of the intact allele and it was prepared a catalog of 22q11.2 hemizygous variation that could be used as a blueprint for future experiments to correlate 22q11.DS variation with the phenotype in the Caucasian population. These authors provided insight into the phenotypic contributions of some genes in the region, but in our study, none of the variants reported were identified. Importantly, since variant frequencies vary across populations, in our study, because we have a Brazilian sample, we also chose to use the Brazilian variant database ABraOM in the process of filtering and interpretation of the variants [22, 59]. One of the limitations of our study is the lack of functional assays performed on the large number of variants detected and the possibility that some variants that did not pass the filter pipeline could affect the phenotype. Secondly, other genes not targeted in this study may be responsible for the 22q11.2DS phenotypic variability. Finally, target NGS data processing methods are limited in detecting genomic structural variants (partial gene deletions or duplications) that have been implicated in the pathogenesis of 22q11.2DS. Despite these limitations, we provide relevant information about the genetic variants found in our cohort that may merit further studies to clarify the phenotypic heterogeneity in 22q11.2DS.

## 5. Conclusions

In conclusion, we performed targeted NGS in a cohort of 60 22q11.2DS Brazilian patients to investigate variants that could act as genetic modifiers. We identified six variants with possible deleterious effects in the context of the 22q11.2 deletion distributed in three genes: *MAPK1, JAM3,* and *ZFPM2*. These variants and genes could be related to cardiac malformations and immune-psychiatric alterations, both phenotypes present in 22q11.2DS. Moreover, the same variants could be identified in more than one patient, suggesting that the co-occurrence of two or more rare variants may have an additive or synergistic deleterious effect with the deletion in 22q11.2. To the best of our knowledge, this study was the first to apply a designed NGS target panel of 22q11.2DS-associated genes that include genes from the region deleted and outside the region, performed in a Brazilian population sample. Nevertheless, the studies that associated the selected genes with the phenotypes studied were carried out in the majority of the Caucasian population, highlighting the relevance of studying their association in the Brazilian population with 22q11.2DS.

## Figures and Tables

**Table 1:** Genes curated for the NGS-panel.

Gene	Position	Ref seq. no.	OMIM	Association
*HIRA*	22q11.21	NP_003316.3	600237	Cardiac anomalies
*CRKL*	22q11.21	NP_005198.1	602007	Cardiac anomalies
*MAPK1*	22q11.22	NM_138957.3	176948	Cardiac anomalies
*HDAC1*	1p35.2	NP_004955.2	601241	Cardiac anomalies
*ZFPM2*	8q23	NP_036214.2	603693	Cardiac anomalies
*JAM3*	11q25	NP_116190.3	606871	Cardiac anomalies
*TANGO2*	22q11.21	NP_690870.3	616830	Immunopsychiatric disorders
*ZDHHC8*	22q11.21	NP_037505.1	608784	Immunopsychiatric disorders
*PI4KA*	22q11.21	NP_477352.3	600286	Immunopsychiatric disorders

**Table 2:** Identified SNVs and their predictions in the present sample (*n* = 60).

Variant Nomeclature^*∗*^	NG_023054.2: g.109490C > T	NM_002745.5: c.^*∗*^1037	NM_002745.5: c.^*∗*^310	NC_000011.10: 134150005: T: C
Gene	*MAPK1*	*MAPK1*	*MAPK1*	*JAM3*
Position (hg19)	22: 22117480	22: 22117502	22: 22118229	11: 134019901
dbSNP (b151)^*∗∗*^	rs897688340	rs13058	rs41282607	rs7936421
MAF (AbraOM)	Not identified in Brazillian population	0.050296	Not identified in Brazillian population	0.153285
MAF (gnoMAD)	0.00000	0.050024	0.021495	0.162251
Location	UTR3	UTR3	UTR3	UTR3
CADD	32	31	24.3	17.07
FATHMM-XL	0.888171	0.887487	0.779486	0.803652
RegulomeDB	Likely to affect binding	Likely to affect binding	Less likely to affect binding	Likely to affect binding
SIFT	NA	NA	NA	NA
eQTLS e GTEX	NA	Artery tibial	Stomach/thyroid	Artery aorta/muscle skeletal
GRASP/trait	NA	Yes/microalbuminuria	NA	Yes/aortic valve
ORegAnno	NA	Transcription factor binding site	Transcription factor binding site	NA
miRNA-target	NA	NA	MAPK1: miR-217	NA
Patients	1 with cardiac malformation	1 with immuno/psychiatric alteration	1 with cardiac malformation	16 with variable phenotype

^*∗*^According to the Human Genome Variation Society; ^*∗∗*^Variant ID from dbSNP (b151); MAF: minor allele frequency, SIFT score: a score <0.5 is considered deleterious; Combined Annotation Dependent Depletion (CADD): a score of 20 means that a variant is amongst the top 1% of deleterious variant; FATHMM-XL: 0 to 1. Scores nearer 1 are more likely to be deleterious.

**Table 3:** Identified indels and their predictions in the present sample (*n* = 60).

Gene	*ZFPM2*	*JAM3*
dbSNP^*∗*^	rs199956937	rs3216140
Position (hg19)	chr8: 106816289 C > CTT	chr11: 134014673 C > CCT
MAF (AbraOM)	Not identified in Brazilian population	0.032787
MAF (gnoMAD)	0.005	0.00003207
Genecanon	Damage	Damage
FATHMM-indel	Damage	Damage
SIFT-indel	09% closer to exon	42% closer to exon
RegulomeDB	NA	Binding site of transcription factor EZH2^*∗∗*^
miRNA-target	miR-130-3p, miR-17-5p, miR-142-5p, miR-340-5p	NA
Patients	3 patients with congenital cardiac malformations	40 patients
Conclusion	Possibly pathogenic	Possibly pathogenic

^*∗*^Variant ID from dbSNP (b151); MAF: minor allele frequency; ^*∗∗*^ChiP-Seq cluster from ENCODE with motifs.

## Data Availability

The genome data generated during this project will be made available upon request. Due to the sensitive nature of genomic information and in accordance with ethical guidelines, access to the data will be granted solely by contacting the corresponding author. Requests for the genomic data should include a brief description of the purpose and intended use of the data, along with the necessary assurances of data privacy and confidentiality. The corresponding author will assess the requests on a case-by-case basis and, if approved, provide the necessary data access and guidance to ensure its appropriate utilization.

## Supplementary Materials

Supplementary Materials Figure S1: Prediction tools and scores used for pathogenicity prediction of variants. Table S1: Variants detected by the NGS panel before filtering. Table S2: Overall coverage per target gene of the NGS panel. Figure S1 shows the different prediction tools used for pathogenicity prediction of the different types of variants found on the patients. Missense variants with CADD >20, FATHMM >0.8, and SIFT <0.05 were considered possibly pathogenic. For nonmissense variants the same scores for CADD and FATHMM were used, as well as the RegulomDB score. For indels, we used GeneCanon and FATHMM-XL scores for pathogenicity prediction. Table S1 shows all the 2,923 variants identified in the nine target genes before filtering application. Location and identification of the variants are reported, as well as the reference and altered sequences. Table S2 shows the overall coverage per target gene of the NGS panel. All genes had an overall coverage >85%. Chromosomal location, number of amplicons, total of bases, total of covered, and missed bases and number of exons for each gene are also reported.
