# Supplementary material for: Variants in Candidate Genes for Phenotype Heterogeneity in Patients with the 22q11.2 Deletion Syndrome
Source: Genet Res (Camb). 2024 Mar 30;2024:5549592. doi: 10.1155/2024/5549592 (PMC10998724; doi:10.1155/2024/5549592)
Supplement: Supplementary Materials — Figure S1: Prediction tools and scores used for pathogenicity prediction of variants. Table S1: Variants detected by the NGS panel before filtering. Table S2: Overall coverage per target gene of the NGS panel. Figure S1 shows the different prediction tools used for pathogenicity prediction of the different types of variants found on the patients. Missense variants with CADD >20, FATHMM >0.8, and SIFT <0.05 were considered possibly pathogenic. For nonmissense variants the same scores for CADD and FATHMM were used, as well as the RegulomDB score. For indels, we used GeneCanon and FATHMM-XL scores for pathogenicity prediction. Table S1 shows all the 2,923 variants identified in the nine target genes before filtering application. Location and identification of the variants are reported, as well as the reference and altered sequences. Table S2 shows the overall coverage per target gene of the NGS panel. All genes had an overall coverage >85%. Chromosomal location, number of amplicons, total of bases, total of covered, and missed bases and number of exons for each gene are also reported. [file 5549592.f1.zip › figure S1.docx]

CADD > 20

FATHMM > 0.8

SIFT < 0.05

SNV *missense*

CADD > 20

FATHMM > 0.8

RegulomDB

SNV *non* *missense*

*indel*

GeneCanon

FATHMM-XL > 0.8

**Supplementary Figure 1. Variants interpreted as deleterious in at least three prediction tools were considered possibly pathogenic**
